# Correlation of the Diagnostic Accuracy of Dual Energy Computed Tomography and Positron Emission Tomography/Computed Tomography in Mediastinal Lymph Node Staging in Lung Cancer: A Systematic Review

**DOI:** 10.7759/cureus.115779

**Published:** 2026-09-04

**Authors:** David Adebayo, Andrew England, Mark McEntee, David J Ryan, Michael Maher

**Affiliations:** 1 Department of Radiography and Radiation Therapy, University College Cork, Cork, IRL; 2 College of Medicine, University College Cork, Cork, IRL; 3 Department of Radiology and Medical Imaging, Cork University Hospital, Cork, IRL

**Keywords:** dect, fdg-pet/ct, lung cancer, mediastinal lymph nodes, staging

## Abstract

This study aims to correlate the accuracy of derived parameters of dual-energy computed tomography (DECT) against the reference imaging modality of choice, positron-emission tomography/computed tomography (PET/CT), in the diagnosis and staging of mediastinal lymph nodes in lung cancer. An extensive database search was conducted to retrieve literature comparing the performance of DECT parameters against PET/CT. Articles published between 2010 and January 2025 were retrieved from Embase, Web of Science, PubMed/MEDLINE (Medical Literature Analysis and Retrieval System Online), CINAHL (Cumulative Index to Nursing and Allied Health Literature), and Ovid. Articles were screened and selected based on clearly defined inclusion criteria. Methodological quality, heterogeneity, and bias were evaluated, and data were extracted and synthesized to identify correlations between DECT parameters and PET/CT parameters in mediastinal lymph node staging. Five articles that met the inclusion criteria were included in this review; these consisted of 212 patients, with 314 pathological mediastinal lymph nodes. DECT attenuation parameters including arterial phase/delayed phase ratio (A/D ratio) or maximum iodine related attenuation (IRA_max_) were either strongly or moderately correlated with maximum standard uptake value (SUV_max_) in PET/CT (r=0.80, p =0.027; r = 0.57, p= 0.047), and iodine concentration or iodine density had a weak or no correlation with SUV_max_ (r=0.23, p=0.14; r=0.064, p =0.453). ^18^fluorine-fluorodeoxyglucose (^18^F-FDG) PET/CT is the imaging modality of choice in the diagnosis of suspected mediastinal lymph nodes, but it is not widely accessible and has higher cost and high false positive rates. This review investigated the potential role of DECT as a possible surrogate of ^18^F-FDG PET/CT and found a good correlation between DECT attenuation parameters (only) and SUV_max_ in ^18^F-FDG PET/CT. There is a significant gap in the literature in relation to the investigation of this correlation. This review found that in DECT, attenuation parameters either strongly or moderately correlated with SUV_max_ from ^18^F-FDG PET/CT and may serve as an excellent complement; however, it may be inadequate to replace ^18^F-FDG-PET/CT in mediastinal lymph node staging in lung cancer.

## Introduction and background

The correct identification of nodal (N)-stage in lung cancer is problematic but essential for prognosis and selection of the best treatment option [1]. In lung cancer, up to 47% of patients with lymph node metastasis have a survival rate of less than five years [2].

Video-assisted thoracoscopic surgery (VATS) (sensitivity 86%, specificity 100%) and endoscopic ultrasound-guided transbronchial needle aspiration (EUTNA) (sensitivity 84%, specificity 100%) can be used for accurate diagnosis; however, these are invasive procedures [3]. Non-invasive imaging modalities are also available for lymph node staging in lung cancer. Currently, ^18^fluorine-fluorodeoxyglucose (^18^F-FDG) positron emission tomography/computed tomography (PET/CT) (sensitivity 77%, specificity 90%) is the reference imaging standard used for this purpose. However, PET/CT has a high false positive rate (up to 55% reported) due to its inability to differentiate reactive non-metastatic lymph nodes (e.g., in tuberculosis or sarcoidosis) from malignant lesions. Additionally, PET/CT is limited by high cost, low availability, and longer acquisition time [4,5]. ^18^F-FDG PET/CT parameters, including maximum standard uptake value (SUV_max_), mean standard uptake value (SUV_mean_), and maximum standard uptake length (SUL_Max_), have been shown to be of value in lung cancer staging, treatment planning, and treatment response assessment [6]. In particular, SUV_max_ has been shown to correlate with tumour aggressiveness and therefore has a predictive value for prognosis [7]. In conventional computed tomography (CT) or magnetic resonance imaging (MRI), morphological features including lymph node size criteria of short-axis diameter and ratio of short-axis to long axis diameter (sensitivity 55%, specificity 81%) have limited diagnostic accuracy due to the possibility of enlargement being caused by micro-metastasis or inflammatory lesions [8].

Dual energy CT (DECT) is a relatively recent imaging modality used in lung cancer diagnosis, staging, and treatment response assessment. Calcification or higher CT attenuation when compared to the great vessels (on non-contrast imaging) is one of the key diagnostic parameters to determine non-malignancy in thoracic lymph nodes [9]. The dual tube voltage setting obtained with DECT acquisitions allows for easy identification of calcified benign lung nodules due to increased X-ray attenuation at lower tube voltages in structures with high effective atomic number (Z) (e.g., calcification or iodinated contrast) caused by the photoelectric effect [10]. In addition, DECT-derived parameters such as iodine concentration (IC), electron density (ED), Iodine enhancement (IE), and normalized iodine concentration (NIC) have been used to quantify tumour perfusion and therefore tumour vascularity, and they are known predictors of prognosis [11].

Some authors have suggested that DECT-based iodine quantification might be an alternative to 18F-FDG PET/CT in patients with non-small cell lung cancer (NSCLC) in the evaluation of treatment response and predicting outcome [12]. As iodine uptake and perfusion parameters correlate, it is plausible to consider that many DECT and ^18^F-FDG PET/CT uptake parameters may also correlate [13]. However, a scoping review of the literature revealed that no systematic review has been performed to examine this possibility.

Therefore, the aim of this review is to systematically assess all literature that compares DECT with ^18^F-FDG PET/CT in lung cancer mediastinal lymph node staging to assess how derived parameters from the two modalities correlate.

## Review

Methods

This review was registered with PROSPERO (CRD42024609690).

Search Strategy

We conducted a comprehensive literature search on the topic of pre-treatment pathological lymph node diagnosis by comparing the performance of DECT against ^18^F-FDG PET/CT in lung cancer staging. We searched databases for literature from 2010 up to January 2025 using search terms as given in Table 1. Grey literature searches, conference abstracts, and unpublished study searches yielded no additional articles. The Preferred Reporting Items for Systematic Reviews and Meta-Analyses (PRISMA) 2020 guidelines were followed.

**Table 1:** Electronic databases and search terms CINAHL: Cumulative Index to Nursing and Allied Health Literature; EBSCO: Elton B. Stephens Company; MEDLINE: Medical Literature Analysis and Retrieval System Online; MeSH: Medical Subject Headings

Databases	MeSH terms, Boolean Operators, Keywords Adapted?	Structured Search Terms
Web of Science	Yes	Dual-energy CT or dual-energy computed tomography) OR TX dual energy spectral computed tomography AND TI positron emission tomography/computed tomography OR AB fdg pet/ct OR TX 18f-fdg pet/ct AND TX (lung cancer or lung neoplasm or lung tumor or lung adenocarcinoma or pulmonary cancer) OR TX Mediastinal lymph nodes/metastasis AND AB staging of cancer.
Embase	Yes	'Dual energy computed tomography'/exp OR 'dual energy spectral computed tomography') AND ('positron-emission computed' OR ('positron emission'/exp OR 'positron emission') AND computed)/exp OR 'pet-ct scanner') AND ('lung cancer staging' OR ('lung'/exp OR lung) AND ('cancer'/exp OR cancer) OR 'metastasis'/dm OR 'neoplasm'/AND ('staging'/exp OR staging).
Scopus	Yes	TI Dual-energy ct or dual-energy computed tomography) OR TX dual energy spectral computed tomography AND TI positron emission tomography/computed tomography OR AB fdg pet/ct OR TX 18f-fdg pet/ct AND TX (lung cancer or lung neoplasm or lung tumor or lung adenocarcinoma) OR TX Mediastinal lymph nodes AND AB staging of cancer.
CINAHL plus full text	Yes	Dual-energy CT or dual-energy computed tomography ) OR TX dual energy spectral computed tomography AND TI positron emission tomography/computed tomography OR AB fdg pet/ct OR TX 18f-fdg pet/ct AND TX ( lung cancer or lung neoplasm or lung tumor or lung adenocarcinoma ) OR TX Mediastinal lymph nodes metastasis AND AB staging of cancer.
Medline/EBSCO host	Yes	AB (dual energy CT or dual-energy CT or dual energy computed tomography or dual-energy computed tomography) OR TI Dual-energy CT OR TX (dect) AND AB positron emission tomography/computed tomography OR TI (pet/ct or positron emission tomography computed tomography) OR TX 18f-fdg pet/ct AND AB (lung cancer or lung neoplasm or lung tumor or lung adenocarcinoma OR TX thoracic neoplasm) OR TI lung cancer staging OR TX Mediastinal lymph nodes staging.
PubMed	Yes	Dual energy CT or dual-energy CT or dual energy spectral computed tomography or dual-energy computed tomography) OR TI Dual-energy CT OR TX (dect) AND AB positron emission tomography/computed tomography OR TI (PET/CT or positron emission tomography computed tomography) OR TX 18f-fdg PET/CT AND AB (lung cancer or lung neoplasm or lung tumor or lung adenocarcinoma OR TX thoracic neoplasm) OR TI lung cancer staging OR TX Mediastinal lymph nodes staging.
OVID	Yes	Dual-energy CT or dual energy spectral computed tomography or dual-energy computed tomography) OR TI Dual-energy CT OR TX (dect) AND AB positron emission tomography/computed tomography OR TI (PET/CT or positron emission tomography computed tomography) OR TX 18f-fdg pet/ct AND AB (lung cancer or lung neoplasm or lung tumor or lung adenocarcinoma OR TX thoracic neoplasm) AND TI lung cancer staging OR TX Mediastinal lymph nodes staging.
Cochrane	Yes	TI Dual-energy CT or dual-energy computed tomography) OR TX dual energy spectral computed tomography AND TI positron emission tomography/computed tomography OR AB fdg pet/ct OR TX 18f-fdg PET/CT AND TX (lung cancer or lung neoplasm or lung tumor or lung adenocarcinoma) OR TX Mediastinal lymph nodes metastasis AND AB staging of cancer OR “TNM* adj3 OR Diagnost*

Study Selection and Inclusion Criteria

A structured selection approach was adapted using the Covidence software (Veritas Health Innovation Ltd, Melbourne, Australia), with two authors individually and then jointly screening the articles. First, title and abstract screening against the inclusion criteria was conducted; if there were discrepancies, these were discussed between the authors and agreement reached by consensus to move the article forward for full-text screening or to reject it. Second, the full-text screening was then conducted in a similar fashion, and selected articles were then moved forward for data extraction. The inclusion criteria adopted for this review are itemised in Table 2.

**Table 2:** Inclusion and exclusion criteria DECT: dual-energy computed tomography; PET: positron-emission tomography; CT: computed tomography

Inclusion Criteria	Exclusion Criteria
English language publication.	Non-compliance with inclusion criteria
Peer-reviewed study, primary research, case-controlled trials, randomized-controlled trials, Quasi-experimental research published in reputable, non-predatory journals.	Case studies and systematic literature reviews
Published between 2010 and January 2025.	-
Be a study comparing DECT with PET-CT in thoracic/mediastinal lymph node and Lung Cancer staging; all lung cancer types were included: non-small cell lung cancer (NSCLC), small cell lung cancer (SCLC), and large-cell neuro-endocrine cancer (LCNEC).	-
Reporting at least one pathologic node per study.	-
Report data for accuracy of the modalities, including sensitivity, specificity, negative predictive values, positive predictive values, odds ratios, and other measures of performance.	-

Data Extraction and Quality Assessment

The methodological quality of the included studies was assessed using a specific tool which addressed different aspects of each study. The Quality Assessment of Diagnostic Accuracy Studies 2 (QUADAS 2) checklist [14] was used to assess for ‘risk of bias’ and the ‘applicability’ questions. General study characteristics and other relevant data were extracted using the Covidence software. There were a limited number of articles found that discuss a comparison between DECT and PET-CT in terms of lymph node staging in lung cancer. Moreover, within those studies selected, there was significant heterogeneity in the approach to comparing DECT with PET-CT, and adequate data could not be retrieved from these studies to perform a good statistical analysis. However, narrative synthesis of the selected studies was performed.

Results

Search and Selection

Database searches yielded an initial total of 31 studies; these were screened against the inclusion and exclusion criteria, and five studies [3,6,7,15,16] that met the eligibility criteria were selected for this review (Figure 1).

**Figure 1:**
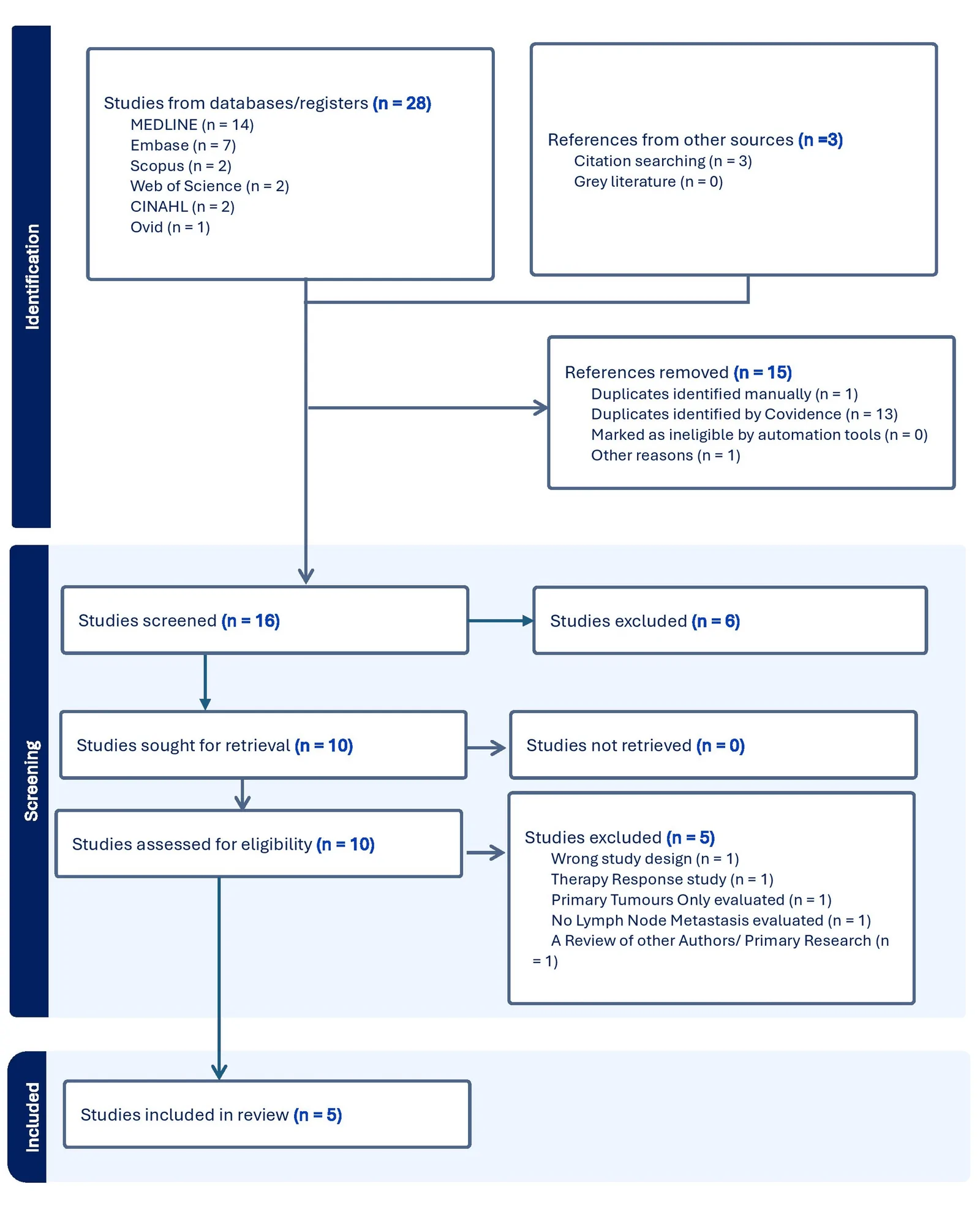
PRISMA Flowchart of Selected Studies PRISMA: Preferred Reporting Items for Systematic Reviews and Meta-Analyses

Quality Assessment and Data Extraction

The methodological quality of included studies was assessed with the QUADAS 2 tool, comprising the “Risk of bias” domain and “Applicability” domain using appropriate signaling questions (Figure 2).

**Figure 2:**
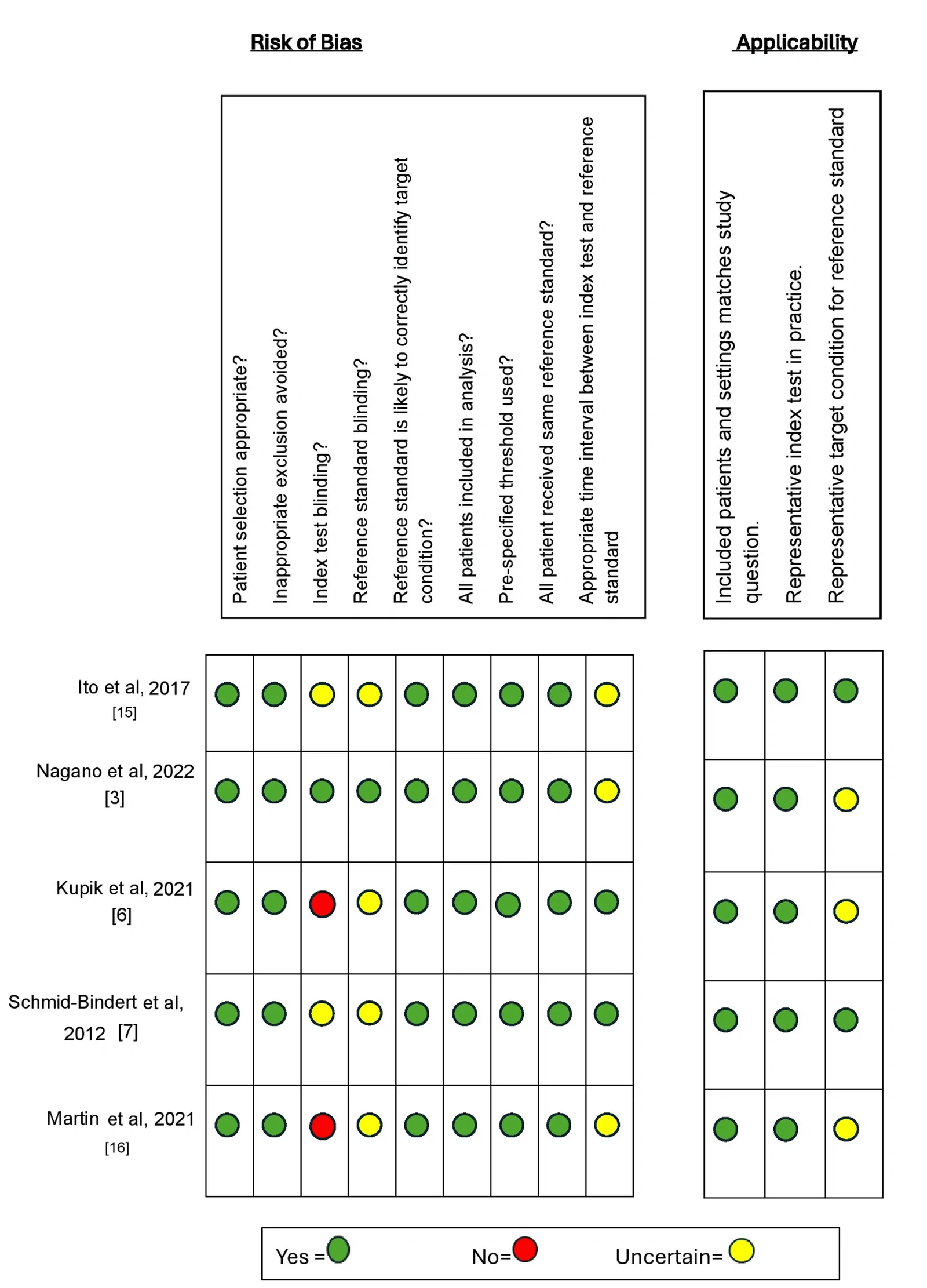
QUADAS 2 diagnostic accuracy checklist QUANDAS 2: Quality Assessment of Diagnostic Accuracy Studies 2 [14]

Overall, selected studies were of fair methodological quality. However, important differences among selected studies include ‘blinding of readers’, time interval between the index test (DECT) and the reference imaging standard (PET/CT), and whether the target condition studied is representative of/applicable to the general population for the reference standard. Four out of five studies did not blind the radiologists [6,7,15,16]. However, blinding of radiologists may not be critical in this clinical setting because it may be necessary for them to ascertain/confirm (anatomically) that the lymph node(s) identified on the DECT image frame being viewed correspond with those being identified on the reference imaging standard at the same level. Additionally, in a typical lung cancer management pathway, patients are referred to PET/CT only after a lesion has been identified on their initial CT scan; therefore, correlation of that lesion on both imaging modalities may be crucial. Thus, not blinding radiologists in these cases may not significantly increase the risk of bias.

In three of the five studies, the time interval between DECT and PET/CT was stated to be less than 28 days [6,7,15]; this is to avoid any significant changes in lesion size, morphology, and characteristics that might affect results when the two modalities are compared [17]. In two studies, this was not discussed [3,16]. In three of the five studies, only NSCLC patients were selected [3,15,16]. Literature highlights that 85% of lung cancer cases are of NSCLC aetiology [18]; however, for those studies in this review that selected only NSCLC cohort, the sample cannot be defined as truly representative of the general population as they have excluded other histologic types which may be significant. Kupik et al. excluded three large cell neuro-endocrine cancer (LCNEC) patients and a large number of lymph nodes with SUV_max_ >2.5 based on clinical experience and expert consensus (judging that relatively high FDG uptake may not specify malignancy) but did not specify their own threshold measurement [6].

Characteristics of Included Studies

The five selected studies were published between 2012 and 2022 [3,6,7,15,16]. There is heterogeneity between the selected studies in terms of design, histology of primary tumour selected, index test protocol, index test parameters used for comparison, and the diagnostic accuracy or correlation values reported.

Patient selection: Four studies selected their patient cohort retrospectively, and one selected them prospectively. There was a total of 212 patients selected overall (179 male, 33 female), with an average age of 66.8 years (range 41-92).

Primary tumour histology: In three studies, only patients with NSCLC were selected [3,15,16], while one selected patients with both NSCLC and small cell lung cancer (SCLC) [7] and one selected all types of lung cancer [6].

Mediastinal lymph nodes: In total, there were 314 pathologic lymph nodes identified in the 212 patients selected. All studies stated the number of pathologic lymph nodes identified by DECT in their patient cohort; however, only two studies stated the number of non-pathologic lymph nodes identified in the same cohort [3,15]. The largest number of pathologic lymph nodes per study was 143 in the study by Kupik et al.; however, they excluded three of these lymph nodes identified as LCNEC from their analysis [6]. Three studies stated the mean lymph node short-axis and long-axis diameter in their cohorts [3,15,16], but only Nagano et al. included these lymph node diameters in their analysis of outcome measures, stating that metastatic lymph nodes exhibited greater short-axis and long- axis diameter than non-metastatic ones (mean short-axis diameter 9.5 mm (metastatic) Vs 6.9mm (non-metastatic); mean long-axis diameter 14.3 mm (metastatic) Vs 12.3 mm (non-metastatic)), and also reported the diagnostic performance of metastatic lymph nodes compared with non-metastatic lymph nodes at short-axis diameter threshold of 8.5 mm (sensitivity 54.5%, specificity 85.7%, accuracy 76.9%) and long-axis diameter threshold of 13.0 mm (sensitivity 63.6%, specificity 73.8%, accuracy 70.9%) [3].

DECT protocol: In general, all studies used late arterial phase CT scans of the thorax to assess thoracic/mediastinal lymph nodes. However, the techniques and timing differ considerably, with delay timing ranging from 30s to 50s after the initiation of contrast media injection. This late arterial phase enables the visualization of thoracic vessels, hilar and mediastinal lymph nodes, primary tumours and nodules. Two of the five studies performed additional delayed scans of the same coverage with a timing delay of 120s and 300s, respectively, to examine for necrosis within the lymph node, distant metastasis, and lymphatic invasion [3,15]. Pathologic lymph nodes were 15% more necrotic than non-pathologic ones.

Contrast media: All studies used non-ionic contrast media injections with volume ranging from 80 ml to 150 ml and concentration varying from 300 mg/ml to 400 mg/ml, which may influence the accuracy of iodine quantification in DECT [19].

DECT post-processing: All studies measured iodine concentration or density using the iodine maps. In addition, four of the five studies used low keV (40 keV) or virtual monochromatic (VM) images to measure iodine attenuation/enhancement [3,6,7,15]. Other DECT post-processing images used include conventional 120 kVp or virtual 120 kVp images, electron density (ED) maps, and effective atomic number maps.

Diagnostic accuracy: Only one study detailed the diagnostic accuracy measures of all the DECT and 18-FDG PET/CT parameters considered [3]. All other studies stated the correlation and p-values between DECT and 18-FDG PET/CT parameters. One study stated the odds ratio only [15].

The characteristics of the five included studies are summarized in Table 3.

**Table 3:** Charcteristics of included studies DECT: dual energy computed tomography; LN: lymph node; PET/CT: positron emission tomography/computed tomography; NSCLC: non-small cell lung cancer; AC: adenocarcinoma; SCC: squamous cell cancer; VMI: virtual monoenergetic image(s); VNC: virtual non-contrast; IRA: iodine related attenuation; ED: electron density; Z: effective atomic number

Arthor	Year	Design	Population (M/F)	Age (years)	Primary Tumour (Hist.)	Number of Pathological (non-Pathological) LNs	LN Axis Diameter (mm), median/ threshold	DECT model	DECT protocol	Contrast media (dose and rate)	DECT post processing	PET/CT model	18F-FDG-dose	Diagnosis accuracy data
Ito et al. [15]	2017	Retrospective	63 (37/26)	Mean 69 (range, 44 -85)	NSCLC Only	14 (not stated)	Not stated	SOMATOM Definition Flash (Siemens Healthineers AG, Erlangen, Germany)	Two phases, arterial bolus track +30s, Delayed -120s	Optiray 320/lopamiron 370 - 96mls @4ml/s	Mean 3D -IRA + 3 material ecomposition algorithm	Biograph 16 (Siemens Healthineers AG)	3.7 mBq/kg or 4.0 mBq/kg	Not given; only OR stated
Nagano et al. [3]	2022	Retrospective	57 (36/21)	Mean 64.8 ± 8.9	NSCLC only	117 (33/84)	>5 mm	Philips IQon (Koninklijke Philips N.V., Amsterdam, Netherlands)	Two phases, arterial bolus track + 50s, Delayed - 300s	(No name) Non-ionic, 2 mls/kg = max 150 mls (rate not stated)	120 kVp, 40 KeV, iodine maps, ED maps, Z maps.	Discovery ™ MI Dr PET/CT (GE Healthcare Technologies, Inc., Chicago, Illinois, United States)	238 mBq	ED: Sensitivity =87.9%, specificity = 58.3%, Accuracy=66.7%
Kupik et al. [6]	2021	Retrospective	68 (65/3)	Median 63.5 (range, 48 - 87)	NSCLC +SCLC	140 (not stated)	15 mm (7 -43 mm)	Discovery ™ CT750HD (GE Healthcare Technologies)	One phase (late arterial, 50 s delay)	(No name) Non-ionic, 80 - 100mls @3-4ml/s	Iodine maps, 40 keV maps (VMI)	Biograph (Siemens Healthineers AG)	3.7 mBq/kg	Not given; only correlation stated
Schmid-Bindert et al. [7]	2012	Retrospective	37 (27/10)	Mean 69 (range, 41 - 92)	All types	86 (not stated)	Not stated	SOMATOM Definition (Siemens Healthineers AG)	One phase (late arterial -35s delay)	lomepro 400/Imeron 400, 150 mls (rate not stated)	IRA, VNC, Virtual 120kVp.	Biograph mCT (Siemens Healthineers AG)	5 mBq/kg	Not given; only correlation stated
Martin et al. [16]	2021	Prospective	21 (14/7)	Median 62	NSCLC	41 (57)	>10 mm	TwinBeam DECT (Siemens Healthineers AG)	One phase, (timing not stated)	Iohexol, Omnipaque 300, 100 mls @3ml/s	Iodine maps	Biograph mCT (Siemens Healthineers AG)	5 mBq	Not given; only correlation stated

DECT vs 18F-FDG PET/CT parameters and correlation coefficient

The most common DECT parameters used for comparison are iodine concentration or iodine density (IC, or ID), iodine-related attenuation (IRA) or iodine enhancement (IE), and LN size or diameter, while the most common 18-FDG PET/CT parameter was SUV_max_ (Table 4).

**Table 4:** DECT vs PET/CT comparative parameters and correlation coefficients for metastatic lymph nodes. SUVmax: maximum standard uptake value; Csize: thin section size on CT; 3D-IRA: 3-dimension iodine-related attenuation; ROC: receiver operating characteristic curve; AUC: area under the curve; FDG: fluorodeoxyglucose; nIC: normalised iodine concentration; nIE: normalised iodine enhancement; SUL: standard uptake length; ID: iodine density; ED: electron density; LN: lymph node Reference guide for Pearson (r) correlation coefficient: 0 – 0.1 = no correlation, 0.2 – 0.4= weak correlation, 0.5 – 0.6 = moderate correlation, 0.7 – 0.9 = strong correlation, 1.0 = perfect correlation.

Author	DECT outcome parameters	PETCT outcome parameters	Lymph Node comparative parameters	Statistical measures	Correlation coefficient	Diagnostic accuracy measures	Correlation Outcome(s) (LN)
Ito et al., 2017 [15]	Csize (CT thin slice) A/Dratio (3D-IRA)	SUVmax	SUVmax vs A/Dratio	Mean ± SD, ROC (AUC), p-value	Pearson (r) Univariate logistic regression	OR	A/D ratio strongly correlates with LN mets [OR=0.972 (95%CI 0.947 -0.997), p=0.027]. SUVmax strongly correlates with LN mets [OR= 1.222 (95%CI 1.046 -1.427), p=0.11].
Nagano et al., 2022 [3]	IC, Z, ED, HU (120 kVp), short axis dia., Long-axis dia. Necrosis.	+FDG uptake	ED, HU (120 kVp) Vs +ve FDG. IC vs ED	Mean ±SD, ROC (AUC), p-value	Pearson (r), Kappa, ICC	Sensitivity, Specificity, Accuracy	ED vs IC = No correlation. Lower ED significantly correlates with metast LN (p=0.001). Higher +FDG uptake significantly correlates with metast LN (p=0.001). Accuracy highest for pairwise combination of ED + short-axis diameter (82.9%).
Kupik et al., 2021 [6]	ICmax, ICmean, nICmax, nICmean, IE (HU), nIE.	SUVmax, SUVmean, SULmax, SULmean, SUVpeak, nSUVmax, nSUVmean, nSULmax, nSULmean	All parameters, e.g.: ICmax vs SUVmax, SUVmean, SULpeak. nICmean vs nSUVmean, nSULmean, nSULpeak, Etc.	Mean ±SD, Median (+IQR), p-value.	Pearson (r), Spearman (p).	None stated	No correlation between IC, IE and SUV, SUL or normalized parameters in entire group or subgroup. e.g. ICmax vs SUVmax (r= 0.064, p=0.0453) IE vs SULpeak (r=0.122, p=0.150)
Schmid-Bindert et al., 2012 [7]	IRAmax, IRAmean, VNCmax, VNCmean, Virtual120 kVp	SUVmax	IRAmax vs SUVmax, IRAmean vs SUVmax	Mean ± SD, p-value	Pearson (r)	None stated	In LNs, moderate correlation between SUVmax vs IRAmax (r=0.570, p=0.047), Weak correlation between SUVmax vs IRAmean (r=0.263, p=0.036). No correlation with other DECT parameters.
Martin et al., 2021 [16]	ID	SUVmax	ID vs SUVmax	Mean ±SD, Median (+IQR), Percentages, ROC (AUC), p-value	Pearson (r), Spearman (p)	Not stated	Weak +ve correlation between ID vs SUVmax of LN (p=0.23, p=0.14). Significant difference in SUVmax between pathologic vs non-pathologic LN. (p<0.01). No significant difference in ID between pathologic vs non-pathologic LN (p=0.43)

IRA or IE was either strongly correlated with SUV_max_ [15] (p=0.027), moderately correlated with SUV_max_ [7] (r=0.57, p=0.047), or not correlated at all [6] (r= 0.122, p=0.150). IC or ID had either a weak positive correlation with SUVmax [16] (r=0.23, p= 0.14) or no correlation at all [6,3] (r=0.064, p=0.453). Iodine concentration or uptake was not significantly different between pathologic and non-pathologic lymph nodes (p=0.18, p = 0.43) except in patients with SCC histology subgroup (p<0.001), whereas SUV_max_ in normal lymph nodes was significantly different from SUV_max_ in pathologic lymph nodes (p<0.001). At a threshold of <2.1mg/ml, ID had an area under the curve (AUC) of only 0.55 on the receiver operating characteristic curve (ROC).

Lymph node size or diameter on DECT was compared in two studies for significant difference between pathologic and non-pathologic lymph nodes and correlation with invasiveness [3,15]. Although one study demonstrated that at a threshold of 8.5 mm short-axis diameter, pathologic lymph nodes show a significant difference from non-pathologic lymph nodes with a sensitivity of 54.5% and specificity of 85.7% [3], the other found no difference or correlation of size with lymph node involvement [15].

Other DECT parameters used by authors to identify pathological from non-pathological lymph nodes include ED; pathological lymph nodes show a significantly lower ED than non-pathological lymph nodes (p<0.001), and necrosis, and pathological lymph nodes are significantly more necrotic than non-pathological ones (p<0.001). They also found that ED does not correlate with IC. 

Authors found that the following DECT parameters have no correlation with any 18-FDG PET/CT parameters, and there were no significant differences between pathological and non-pathological lymph nodes; an absence of statistical correlation was observed with effective atomic number (z), IC_mean_, normalized IC or normalized IE, virtual non-monochromatic (VNC)_max_ /mean, virtual 120 kVp, long-axis to short-axis ratio, lymph node shape, or margin.

Diagnostic Performance

Diagnostic accuracy data were supplied by only two studies [3,15], one of which had partial data. Ito et al. (2017) showed that arterial phase/delayed phase attenuation (A/D) ratio significantly correlates with pathological invasiveness (OR =0.973; 95%CI 0.954 - 0.992, p= 0.005) and significantly correlates with LN involvement (OR=0.972; 95%CI 0.947 - 0.997, p=0.027) (Table 5) [15].

**Table 5:** Correlation coefficients and diagnostic accuracy OR: odds ratio; HU: Hounsfield unit; ED: electron density; SUVmax: maximum standard uptake value; SULpeak: peak standard uptake length; IC: iodine concentration; IRA: iodine-related attenuation; ID: iodine density; LN: lymph node; DECT: dual-energy computed tomography; A/D: arterial phase/delayed phase attenuation

Study (author, year)	Specific (key) consideration(s)/ Clinical question	Correlation coefficient	Diagnostic Performance (Accuracy)	Comment/Conclusion
Ito et al., 2017 [15]	DECT: 3D-IRA is corelated with degree of differentiation and locoregional invasiveness. PET/CT: SUVmax is well correlated with tumour malignancy, pathological invasiveness and post-therapeutic prognosis. Therefore, is 3D-IRA (A/D ratio) correlated with SUVmax	A/D ratio strongly correlates with LN mets (OR=0.972; 95%CI 0.947 -0.997), p=0.027. SUVmax strongly correlates with LN mets (OR= 1.222; 95%CI 1.046 -1.427), p=0.11	OR=0.972, p=0.027; OR=1.222, p=0.11, Sensitivity = 65.7%, A/D ratio for specificity=75% invasiveness	A/D ratio significantly correlates with SUVmax for LN metastasis
Nagano et al., 2022 [3]	There is a linear relationship between ED (measured by DECT) and tissue density and composition. ED is useful in predicting vascularity in meningiomas. Does ED (from ED) have utility in diagnosing metastatic lymph nodes in NSCLC compared with conventional CT and FDG PET/CT?	Lower ED significantly correlates with mets LN (p=0.001). Higher +FDG uptake (SUVmax) significantly correlates with mets LN (p=0.001). Accuracy highest for pairwise combination of ED + short-axis diameter (82.9%).	120 kvp (HU) sensitivity = 51.5%, specificity =79.8%, accuracy = 71.8%; ED: sensitivity =87.9%, specivicity = 58.3%, accuracy = 66.7% +FDG uptake (SUVmax): sensitivity = 66.7%, specificity = 75.0%, accuracy = 72.6%	In NSCLC, metastatic LNs exhibited lower ED than non-metastatic LNs. Metastatic LNs exhibit higher +FDG uptake (SUVmax) than non-metastatic LNs. IC not significantly different between metastatic and non-metastatic LNs
Kupik et al., 2021 [6]	IC in DESCT can be used to differentiate primary lung cancer and to discriminate between benign and malignant pulmonary nodules. IE may reflect tumour vascularity. Increased metabolism, assessed by 18F-FDG PET/CT is associated with earlier tumour recurrence. Is there a relationship between DESCT and 18F-FDG PET/CT parameters in primary tumours and metastatic LNs of lung cancer?	No significant correlation between IC, IE and SUV, SUL or normalized parameters in the entire group or subgroup; e.g., ICmax vs SUVmax (r= 0.064, p=0.0453). IE vs SULpeak (r=0.122, p=0.150)	Not stated.	No significant relationship between DESCT and FDG PET/CT for metastatic LNs.
Schmid-Bindert et al., 2012 [7]	DECT can differentiate pulmonary nodules as benign or malignant with a sensitivity of 92%. If iodine distribution (on DECT) in tissue strongly correlates with local blood volume and vascularization, then DECT may be used to assess angiogenesis of tumours and may be used as a surrogate for FDG PET/CT.	In LNs, moderate correlation between SUVmax vs IRAmax (r=0.570, p=0.047), Weak correlation between SUVmax vs IRAmean (r=0.263, p=0.036), No correlation between SUVmax and all other DECT parameters	Not stated.	Moderate correlation between IRAmax and SUVmax for LN involvement. Weak correlation between IRAmean vs SUVmax for LN involvement.
Martin et al., 2021 [16]	ID, iodine quantification (from DECT) has applications in the diagnosis and prognosis of malignancies. Can ID be used as an indirect biomarker of tumour metabolism by comparing DECT ID against PET/CT-derived SUVmax?	Weak +ve correlation between ID vs SUVmax of LN (p=0.23, p=0.14). No significant difference in ID between pathologic vs non-pathologic LN (p=0.43)	Not stated.	Weak +ve correlation between ID vs SUVmax of LNs. Iodine uptake (ID) is not suitable for detection of metastatic LNs

A/D ratio had a sensitivity of 65.7% and specificity of 75.0% as diagnostic performance to determine pathological invasiveness. SUV_max_ significantly correlates with pathological invasiveness (OR= 1.200; 95%CI 1.015 - 1.420, p= 0.033) and significantly correlates with LN involvement (OR= 1.222; 95%CI 1.046 - 1.429, p= 0.011)

Nagano et al. identified the diagnostic performance of the following radiologic parameters: (i) attenuation on 120 kVp (HU) images: sensitivity 51.5%, specificity 79.8%, accuracy 71.8%; (ii) ED: sensitivity 87.9%, specificity 58.3%, accuracy 66.7%; (iii) +ve FDG uptake (SUVmax) sensitivity 66.7%, specificity 75.0%, accuracy 72.6% [3]. They also showed that accuracy was highest when ED was combined with short-axis diameter (sensitivity 54.5%, specificity 94.0%, accuracy 82.9%) and when ED was combined with +ve FDG uptake (sensitivity 60.6%, specificity 90.5%, accuracy 82.1%).

Using FDG PET/CT as a surrogate (/imaging) reference standard, 2x2 contingency tables were constructed with these data, and using appropriate formulas, some calculations were performed to further explore diagnostic performance (using PET/CT as surrogate reference standard implies that reported diagnostic performance requires careful consideration). We found that the A/D ratio has a sensitivity of 65.7%, specificity 75.0%, accuracy 71.9%, OR 5.77, positive predictive value (PPV) 57.4%, negative predictive value (NPV) 81.1%, and LR+ 2.63. Attenuation on 120 kVp has sensitivity 51.5%, specificity 79.8%, accuracy 71.8%, PPV 50.0%, NPV 57.4%, OR 4.1, LR+ 2.55. ED sensitivity 87.9%, specificity 58.3%, accuracy 66.7%, PPV 45.3%, NPV 92.5%, OR 10.2, LR+ 2.09. ED does not correlate with IC or IE (or A/D ratio).

Discussion

This systematic review reveals that DECT has a significant complementary role to play along with 18F-FDG PET/CT in thoracic/mediastinal lymph node staging in lung cancer. There is a significant gap in the literature and limited research data investigating the potential role of DECT as a possible surrogate for 18F-FDG PET/CT in mediastinal lymph node staging, which could bring about significant improvement in the management of patients’ treatment pathway and prognosis. Thoracic/mediastinal lymph node staging is significantly important for the patient as it is a prognostic marker. 18F-FDG PET/CT is currently used as a non-invasive reference imaging standard because of its ability to detect tumour activity even in non-enlarged lymph nodes. However, 18F-FDG PET/CT is unable to differentiate inflammatory disease in lymph nodes from tumoural activity and has a high false positive rate, meaning that there is a potential for a patient to be denied curative surgery if a positive FDG PET/CT result is not further investigated with an invasive mediastinal procedure like endobronchial ultrasound (EUBUS) [20].

There were only five studies found that have attempted to compare the diagnostic performance of DECT to that of FDG PET/CT over a period covering over a decade, and even these were with significant heterogeneity in their methodological design, scanner types, protocols, parameters investigated, analysis performed, and outcome measures. Only one of the five studies was a prospective study [16], and a larger percentage of the studies investigated only NSCLC, raising the question of bias and applicability to the general population, although 85% of all lung cancers are NSCLC. This implies a restriction in the ability to draw a firm conclusion on the correlation between DECT and PET/CT, highlighting the need for further research in this area. However, the general risk of bias is judged as low across all studies selected due to their methodological rigor.

This systematic review found that any measure/parameter of attenuation on DECT (A/D ratio or IRA or attenuation on 120 kVp images) is identified to correlate either significantly or moderately with SUV_max_ on FDG PET/CT, rather than a measure/parameter of iodine content (IC/nIC/ID) or vascularity alone. IC or ID had a weak correlation with SUV_max_ or no correlation at all in the selected studies. This is significant because attenuation is affected by several factors, including concentration of contrast media, tube voltage (kVp), tissue vascularity/viability, tissue cellularity, and matrix density. Higher contrast media concentration may cause increased attenuation due to higher photoelectric absorption, and the lower the iodine concentration, the higher the absolute percentage error when higher tube voltages, e.g., 140 kVp, are used [19]. Iodine enhancement at low kVp (80 kVp) is higher than iodine enhancement at higher kVp (140 kVp), and lymph nodes with higher net contrast enhancement have slightly higher CT attenuation on VNC images than on true non-contrast images [9], and pathological lymph nodes are known to have a lower vessel count (many of which are anaplastic) and more necrotic than non- pathologic lymph nodes [21], which may lead to relatively lower iodine uptake in pathological lymph nodes compared to non-pathologic lymph nodes [22,23]. Similarly, SUV_max _reflects glucose metabolism in tissue, which is influenced by cell density, vascularity, tumour aggressiveness and inflammation, factors which also impact CT attenuation.

This result is supported by several studies. In a meta-analysis by Wang et al., the authors found that a pairwise combination of normalized IC (nIC) and slope of the spectral curve (λHU) in identifying pathologic from non-pathologic lymph nodes was better than short-axis diameter alone [5]. Arterial phase nIC has a sensitivity of 85%, and λHU has a specificity of 84%, and they will complement each other for difficult diagnoses in lymph nodes. Aberrant vessels and micro-infiltrations by cancer cells will cause lumen narrowing and reduced blood supply in pathologic lymph nodes; therefore, nIC in pathologic lymph nodes will be lower than in non-pathologic ones [4]. Similarly, Gao et al. found that arterial enhancement fraction (AEF) had the greatest diagnostic efficiency for metastatic LN among all DECT parameters considered, with a sensitivity of 90.0%, specificity of 89.5%, PPV of 88.9%, and NPV of 90.6% (at 95% CI 0.83 -0.94) [24].

Other DECT parameters investigated in the included studies include Z and ED. While Z was found to have no correlation with SUV_max_, lower ED significantly correlated with metastatic lymph node (p<0.001), and a pairwise combination of ED and short-axis diameter had the highest accuracy at 82.9%. ED correlates well with positive FDG uptake (SUVmax) but had no correlation with IC [3]. ED is routinely used in radiotherapy for radiation dose distribution calculation for therapy planning and treatment optimisation. DECT allows direct calculation of ED (and Z) on a pixel-by-pixel basis, helping to eliminate calibration curve mapping for photon therapy and improve range accuracy for proton therapy [25].

Data to perform adequate meta-analysis and synthesis were generally absent or inadequately supplied in the selected studies. From the data available in selected studies, only parameters of attenuation on DECT show significant correlation with SUV_max_ on PET/CT, with high specificity and negative predictive values. These are, however, from only two of five studies and are grossly inadequate to draw conclusions of correlation or performance.

This systematic review has some limitations. Meta-analysis could not be carried out due to inadequate data. However, even if adequate data to perform meta-analysis were available, the results would have needed to be cautiously considered and interpreted as the number of selected studies is very small (n=5). A larger number of the studies were retrospective, and non-English language publications were also excluded.

## Conclusions

This systematic review reveals that, based on current data, DECT attenuation parameters either significantly or moderately correlate with 18F-FDG PET/CT parameters. DECT may serve as an excellent complement but may be inadequate to replace 18F-FDG PET/CT in mediastinal lymph node staging in lung cancer. Further research is required to investigate this phenomenon.
